# High gain modified Vivaldi vehicular antenna for IoV communications in 5G network

**DOI:** 10.1016/j.heliyon.2022.e09336

**Published:** 2022-04-27

**Authors:** Ankush Kapoor, Pradeep Kumar, Ranjan Mishra

**Affiliations:** a Department of Electronics and Communication Engineering, Jawaharlal Nehru Government Engineering College, Sundernagar, Mandi, India; b Discipline of Electrical, Electronic and Computer Engineering, University of KwaZulu-Natal, Durban, 4041, South Africa; c Department of Electrical and Electronics Engineering, University of Petroleum and Energy Studies, Dehradun, India

**Keywords:** Vivaldi antennas, Tapered slot antennas, Internet of vehicles (IoV), Vehicular antennas

## Abstract

While targeting the sixth generation (6G) wireless communication which incorporates mainly the Internet of Things (IoT), the Internet of Vehicles (IoV) becomes a vital component to be implemented. A vehicular antenna is designed by using the modified design of the Vivaldi antenna that can cover all the frequency bands for the long-term evolution (LTE) and the mid-band fifth-generation (5G) wireless systems (ranging from 3.3 GHz to 6.5 GHz). The proposed design is optimized by analyzing the effect of the variation in the dimensions of the antenna, changing the position of the taper, the rectangular slot dimensions and the diameter of the circular slot etc. The presented automotive antenna operates at four frequency bands ranging from 3.62 GHz - 4.03 GHz (410 MHz), 4.17 GHz–5.38 GHz (1210 MHz), 5.57 GHz–6.16 GHz (590 MHz) and from 6.23 GHz - 6.64 GHz (410 MHz) with the minimum reflection coefficient of -39 dB, -39.06 dB, -50.42 dB and -12.45 dB, respectively. The proposed antenna offers the high gain of 7.6 dB, 6.7 dB, 9.3 dB and 7.4 dB for the four bands of the operating spectrum, respectively. The analysis of the reflection and radiation characteristics of the antenna when placed on the vehicle confirms the suitability of an antenna for the smart vehicle to vehicle communications.

## 1 Introduction

**Figure 1:**
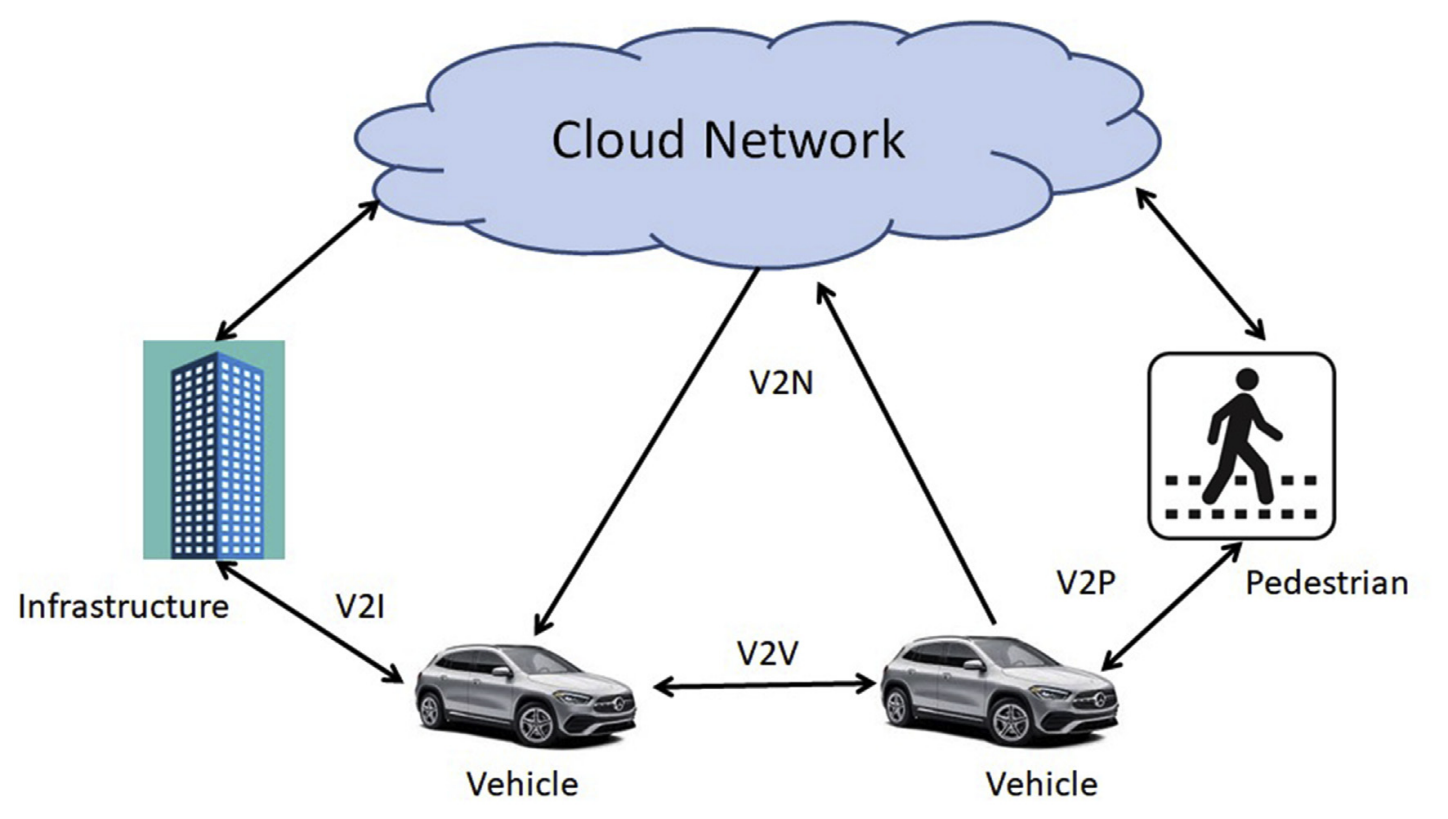
Various V2X connections in IoT systems.

With the advancements in the IoT, the IoV has become a significant aspect of wireless communication systems. There has been an exponential growth in the usage of wireless devices that bring comfort to every person's life. In the latest research, it is seen that the demand for the planar antennas has been increased in the wireless communication devices for catering to the needs of vehicular communications which focuses on Machine-to-Machine (M2M) communications. Automobiles are equipped with a variety of operational antennas that may be used for emergency calls, geo-positioning and mapping to suit the many demands of the modern cellular phones [1]. The design of an automotive antenna is targeted to achieve an increase in the quality of the service (QoS) of the wireless transmission system. In the fifth generation (5G) wireless communications, it is possible to achieve shorter cycle trip delays with reduced energy usage by making use of the IoTs [1]. The IEEE has also upgraded its 802.11 standard to 802.11p for the wireless connectivity in the dedicated short-range for vehicle-to-vehicle (V2V) and vehicle to infrastructure (V2I) systems [2]. Depending on these needs, the IoV system has created an interest of the researchers for its development. The IoTs is a worldwide network system that allows billions of electromechanical devices and electronic gadgets to connect and communicate with each other. To satisfy the various demands of contemporary communication devices, automobiles are outfitted with a variety of functional antennas that may be used for emergency calls, entertainment, navigation, and location. An important vehicle to everything (V2X) scheme that is catching the eye of the researchers is the DSRC which refers to the exchange of information between autos over a short distance by the use of wireless technology. It comprises of the following types of equipment (a) an onboard device (OBU) positioned within the vehicle; (b) a road test unit (RSU) located on the side of the road; and (c) a portable device carried by walkers [3]. V2X is classified into four types: V2V, V2I, vehicle to network (V2N), and vehicle to pedestrian (V2P). Figure 1 depicts V2X situations in future IoT systems.

The V2V network is made up of automobiles that are linked to one another. V2I and V2P are formed when cars connect with structures and pedestrians. The V2N network might be a component of a cellular system when connected remotely to a cloud. Presently, all the planar antennas fall in the two categories of the broad-side and end-fire antennas. Broadside antennas mainly consist of resonant shapes such as dipoles, slots and patches. A major drawback of these antennas is their low gain and narrow bandwidth which provides a great challenge in their usage. The use of the defected ground structure which when integrated with the patch antenna provided an effective technique in improving the radiation properties of the radiating structure [4]. The study is further extended in which it is suggested that the use of the stub loaded patch antenna helps in achieving the miniaturization [5]. The phenomenon of cutting the slots inside a patch antenna is helpful in increasing the bandwidth of the patch antenna at the desired frequency of operation. When this technique is combined with the defected ground structure, then it is possible to get the most efficient structure in the form of wide bandwidth [6]. Various design geometries of the Vivaldi antennas have been reported in the literature consisting of non-linear tapering, linear tapering and maintaining a constant width [7, 8, 9]. Primary features that include wide scanning, enhanced efficiency, unilateral behavior, and the capability of integration within the small chips make these Tapered Slot Antennas (TSA) as the best choice for the vehicular communication. Due to the aforesaid advantages of the TSA, they can be an ideal candidate for the mid-band sub-6 GHz 5G applications such as enhanced mobile broadband and ultra-low latency communications. The first time name of the TSAs in the literature occurred in the year 1979 in the ninth European Microwave Conference by two publications [10]. Applications of the single element TSAs can be seen in the feeders which are used for lens antennas. Due to the inherent advantage of low cost of fabrication, these antennas form the best candidates for low cost integrated transceivers as shown in the research [11, 12]. Vivaldi design of the tapered antenna has shown its importance among researchers due to its inherent property of giving a high gain and wide bandwidth with compact dimensions [13]. Due to such properties, it has become a suitable candidate to be used in radar applications [14]. The patch designed with a slot is placed on the substrate on one side and on the other side ground plane is designed. On visualization of an extreme corner of the design, the slot width becomes narrower which helps in coupling of the radiation effectively to the other active devices such as transistors and diodes [15]. Sub-6 GHz ranges of frequency spectrum are suggested and classed in 5G wireless system improvements. Lower frequency bands (3.3 GHz–6 GHz) have been recommended for 5G applications at the ASEAN fifth-generation (5G) conference, which has inspired in the designing of the antennas for the FR1 range of the 5G spectrum. The applications which are targeted in this research area are the enhanced mobile broadband communication, massive machine type communications and the vehicular communications [16, 17]. Dielectric substrate material which is being used in the antenna design has a great role to play in the design of the microstrip transmission line and the antenna radiating aperture. Some of the important characteristics which must be considered while designing the dielectric substrate are the value of dielectric constant, the value of thermal expansion and conductivity, market rate, availability and the effective thickness of the copper surface [18, 19]. Recently, researchers have oriented their approach towards the design of the high gain wideband planar antennas which must possess the capability to cater an enormous number of users as per the demand of the 5G wireless systems. In the future vehicular networks, the automobiles will be coupled with additional communication devices for accomplishing more extensive and intelligent driving functions. The integration of an antenna into a specific tiny volume model, such as the shark-fin-shaped roof antenna, is a significant problem. Meanwhile, figuring out how to implement a diverse set of services over many frequency bands is a significant challenge. While fixed volume architecture may accommodate many antennas of different frequency ranges, it is important to eliminate antenna coupling and increase radiation efficiency [20]. To eliminate this problem, a novel decoupling and matching network architecture has been illustrated in the literature [21]. A multifunctional microstrip-fed ultra-wideband monopole antenna with the two dual-mode resonators is presented to eliminate the problem of interference [22]. In the literature, a novel planar microstrip-fed monopole ultra-wideband antenna with two notched bands has been described for performing the vehicular communications [23]. Due to the challenge of improving antenna isolation, waveband antennas are being studied extensively for many frequency bands. Different types of automotive antennas for IoT systems have been explored using various methodologies depending on the cost and efficiency of the antenna [24, 25, 26, 27]. The DSRC smart shark-fin antennas are intended for LTE, WLAN, and DSRC communication systems, and have been used in vehicle communication [28, 29, 30]. The multiband antenna is somewhat hard to design and implement on the machine as it requires a high production cost.

In this paper, a new approach of designing the linear tapered slot antennas for operation within the mid-band 5G devices possessing a wider bandwidth and enhanced gain with a large value of directivity is presented. Initially, the bandwidth of the proposed antenna is enhanced by the usage of the microstrip to slot line progression feed. Along with the bandwidth enhancement in the design, the gain is also enhanced by incorporating a rectangular slot in between the circular slot and tapering end of an antenna which helps in providing a guided wave along the desired direction. The proposed end-fire antenna provides a gain of 5–9 dB within the mid-band 5G operating region (3.3 GHz–6 GHz). A simple rectangular strip line coupled to slot in the patch is used in the ground plane which adds to the simplicity in fabrication. The organization of our paper is as follows: Section 2 presents the geometry of the multiband vehicular antenna; Section 3 deals with the interpretation of the results retrieved from the tapered slot antenna; Section 4 describes the variation in the antenna characteristics when incorporated upon the automobile and finally, the work has been concluded in section 5.

## 2 Geometry of the proposed antenna

**Figure 2:**
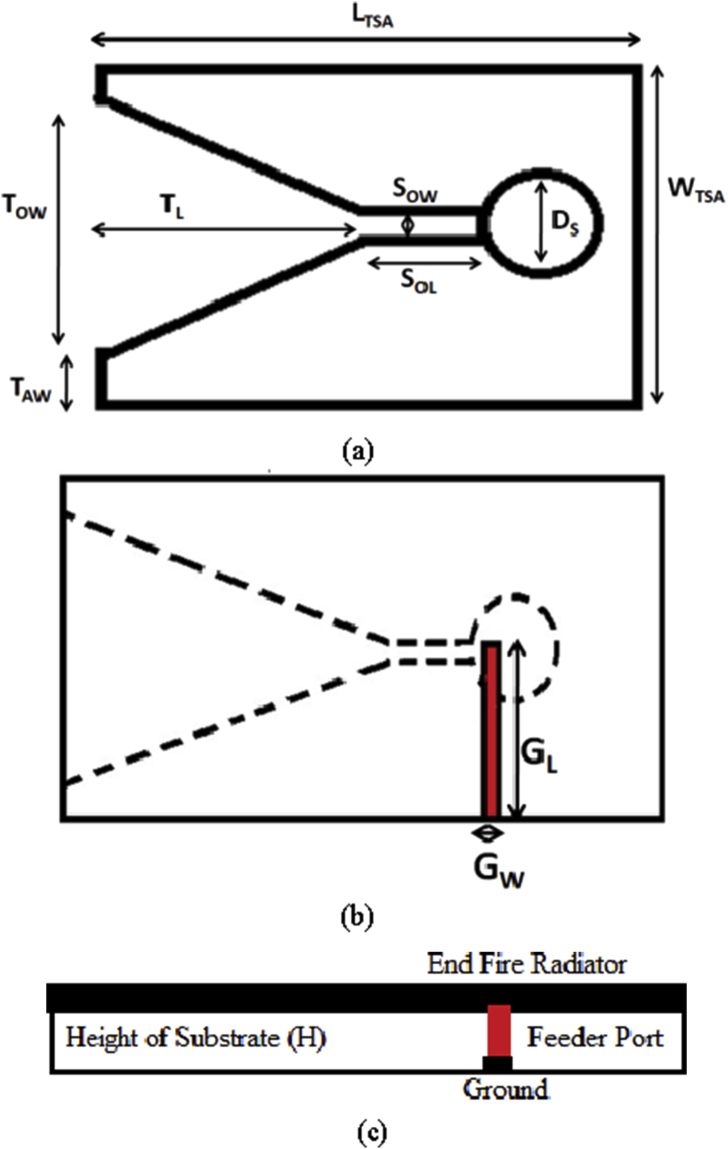
Geometrical configuration of the LTSA, (a) top view, (b) bottom view and (c) side view.

The innovative vehicular communication antenna is supposed to perform in various frequency bands, including all LTE, 5G, WLAN, and DSRC frequencies. The proposed wideband linear tapered slot antenna (LTSA) is discussed based on the electrical properties as shown in the Figure 2. A microstrip to slot line progression feed is utilized for getting accurate results. The geometrical configuration consists of the five parts composed of the radiating patch, circular slot with diameter D_s,_ rectangular slot (S_OL_ x S_OW_), tapered slot and a ground plane with dimensions G_L_ x G_W._ The radiating patch covers an area of L_TSA_ x W_TSA._ This design is made up of a FR4 substrate with a permittivity of 4.4 and a thickness of 1.6 mm.

The antenna parameters contribute a major role in defining the structure and dimensions of the planar LTSA's to be used for the high frequency operations. Some of the parameters consist of the choice of a dielectric substrate, variation of its thickness and permittivity with the frequency. Many other parameters significantly affect the performance of LTSA's such as parameter variations of the ground, thickness of the substrate, dimensions of the slots and the taper angle. The basic design parameters which are required for the designing of LTSAs in the mid-band 5G frequency spectrum are characterized as the length of linear tapered slot antenna (L_TSA_), width of linear tapered slot antenna (W_TSA_), circular slot diameter (D_S_), slot opening width (S_OW_), slot opening length (S_OL_), tapered length (T_L_), tapered antenna width (T_AW_), tapered opening width (T_OW_), substrate height (H), ground width (G_W_), ground Length (G_L_). The design fundamentals required for making the structure of LTSA's are given in the past research, which consist of some basic rules as described in the following Eqs. (1), (2), (3), (4), (5), (6), (7), (8), (9), (10), (11), (12), and (13) [11]:

Width of tapered slot antenna:

(1) $${\text{W}}_{\text{TSA}}\geq {\text{λ}}_{\text{m}}$$

(2) $$1.05\leq \frac{\text{c}}{\text{v}}\leq 1.2$$

Effective thickness of the conducting surface (${\text{t}}_{\text{eff}}$):

(3) $$0.005{\text{λ}}_{\text{m ​}}\leq \left(\sqrt{{\text{ε}}_{\text{r}}}-1\right){\text{t}}_{\text{eff}}\leq 0.03{\text{λ}}_{\text{m ​}}$$

Length of tapered slot antenna:

(4) $$2{\text{λ}}_{\text{m ​}}\leq {\text{L}}_{\text{TSA}}\leq 12{\text{λ}}_{\text{m ​}}$$

Tapered opening width:

(5) $$\text{λ}{\text{T}}_{\text{OW}}>\frac{{\text{λ}}_{\text{m}}}{2}$$

Height of the substrate (H):

(6) $$\text{H ​}\gg 0.003{\text{λ}}_{\text{m ​}}$$

Overall length of the slot line $\left({\text{S}}_{\text{OL}}\right)$ is given as:

(7) $$0.2{\text{λ}}_{\text{m}}\leq {\text{ ​S}}_{\text{OL}}\leq 0.5{\text{λ}}_{\text{m}}$$

Opening width of slot line $\left({\text{S}}_{\text{OW}}\right)$ :

(8) $$0.06{\text{λ}}_{\text{m}}\leq 0.09{\text{λ}}_{\text{m}}$$

Ground length $\left({\text{G}}_{\text{L}}\right)$:

(9) $${\text{G}}_{\text{L}}=\frac{\text{c}}{2{\text{ ​f}}_{\text{r}}\sqrt{{\text{ε}}_{\text{eff}}}}$$

Ground width $\left({\text{G}}_{\text{W}}\right)$:

(10) $${\text{G}}_{\text{W}}=\frac{7.48\text{H}}{\exp\sqrt{0.33\sqrt{{\text{ε}}_{\text{r}}+1.41}}}-1.25{\text{t}}_{\text{eff}}$$

where ${\text{λ}}_{\text{m}}$ is the wavelength of an electromagnetic wave which is occurring at the center frequency (4.8 GHz) when passed through a waveguide or an antenna, c is the velocity of light, v is the velocity of electromagnetic fields within the slot, ${\text{ε}}_{\text{r}}$ is the dielectric constant of the substrate and ${\text{ε}}_{\text{eff}}$ is the effective dielectric constant. Dielectric substrate material used in the antenna design has a great role to play in designing the microstrip transmission line and antenna radiating aperture. Some important dimensions to consider while designing of the dielectric substrate are value of dielectric constant, value of thermal expansion and conductivity, market rate, availability, effective thickness of the copper surface [16]. It is important to mention in our design that the effective dielectric constant (ε_eff_) is different from dielectric constant (ε_r_) of the substrate. The values of both the parameters are equal only in the case of a homogeneous structure and not for non-homogeneous surfaces. The effective dielectric constant lies in between:

(11) $$\frac{1}{2}\left({\text{ε}}_{\text{r}}+1\right)\leq {\text{ε}}_{\text{eff}}\leq {\text{ε}}_{\text{r}}$$

If G_w_ denotes the ground plane width and H denotes the substrate thickness then two cases are defined as:

When ${\text{G}}_{\text{W}}\geq \text{H ​}$, then the circuit behaves as if two parallel lines are running together because most of the electromagnetic fields are concentrated within the microstrip patch. In this case ε_eff_ is approximately equivalent to ε_r._

On the other hand when ${\text{ ​ ​G}}_{\text{W}}\leq \text{H}$, then concentration of the electromagnetic fields is distributed in such a way that fifty percent of the fields are transmitted through the air with ${\text{ε}}_{\text{r}}=1$, while remaining are transmitted through the substrate with ${\text{ε}}_{\text{eff}}=\frac{1}{2}\left({\text{ε}}_{\text{r}}+1\right)$. Further for identifying the precise value of ${\text{ε}}_{\text{eff}}$, we define the following expressions by taking into consideration the negligible thickness of the microstrip patch.

(12) εeff=(εr+1)2+(εr−1)2[(1+12GWH)12+0.04(1−GWH)];∀GWH≤1

(13) εeff=(εr+1)2+(εr−1)2[(1+12GWH)12]∀GWH≥1

The initial dimensions were evaluated from the set of design equations as presented and the parametric sweep option is utilized for getting adequate results at the frequency bands of operation. The values extracted by performing the parametric analyses are depicted in Table 1 as given below:

**Table 1:** Dimensions of the proposed LTSA.

Parameters	Values (λ_m_ denotes the centre wavelength)
Length of tapered slot antenna (L_TSA_)	1.76 λ_m_
Width of tapered slot antenna (W_TSA_)	1.28 λ_m_
Circular slot diameter (D_S_)	0.39 λ_m_
Slot opening width (S_OW_)	0.06 λ_m_
Slot opening length (S_OL_)	0.32 λ_m_
Tapered length (T_L_)	1.02 ​λ_m_
Tapered antenna width (T_AW_)	0.06 λ_m_
Tapered opening width (T_OW_)	1.15 λ_m_
Substrate height (H)	0.024 λ_m_
Ground width (G_W_)	0.080 λ_m_
Ground length (G_L_)	0.612 λ_m_

**Figure 3:**
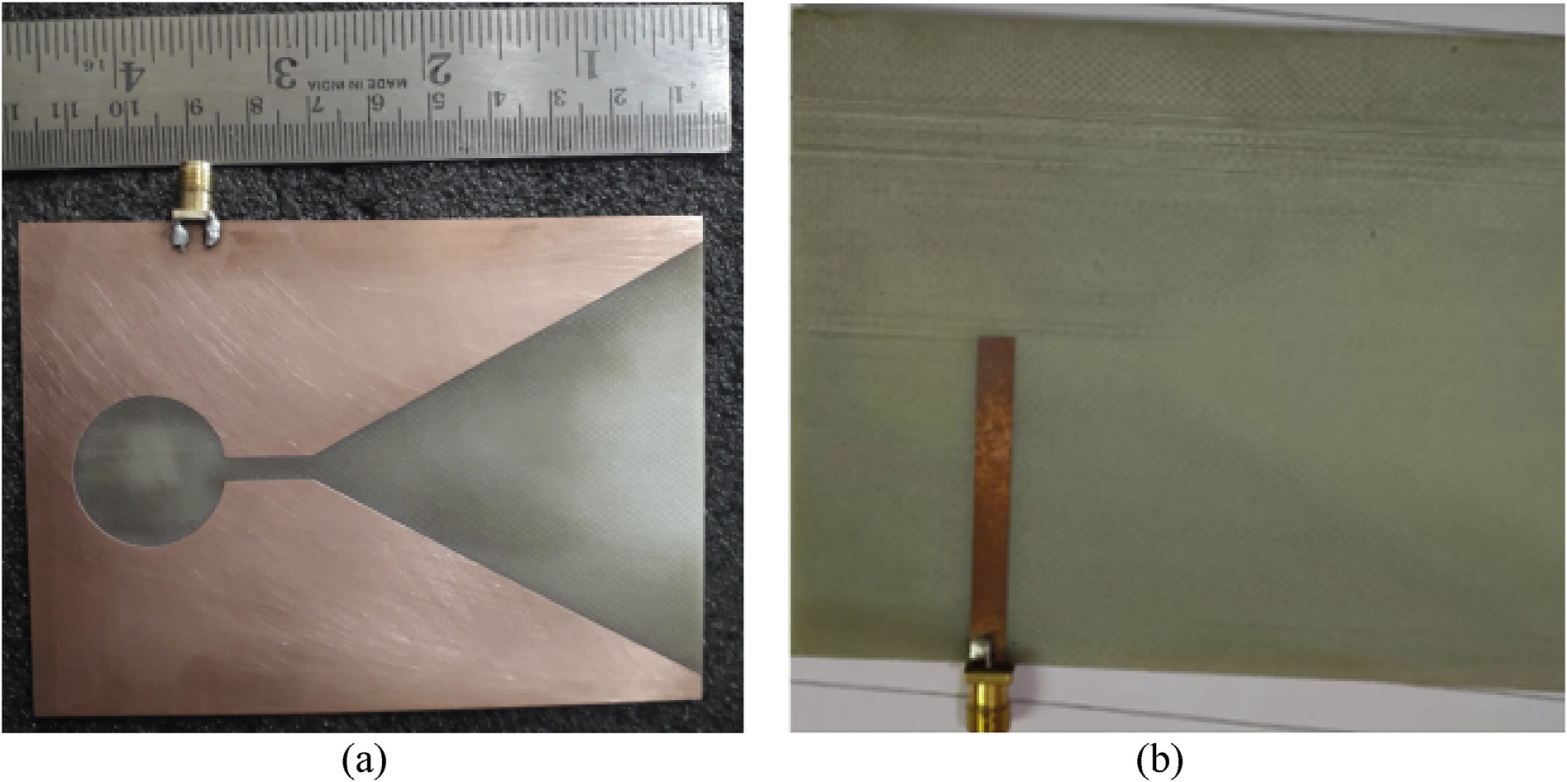
Photographs of the fabricated LTSA, (a) front view, and (b) back view.

The prototype of the proposed antenna is fabricated with the dimensions as discussed in the Table 1 by using the commercially available FR4 substrate and the reflection coefficient is measured by using the proper experimental set-up. The fabricated antenna is depicted in Figure 3.

There are some of the important performance parameters which define the antenna characteristics such as reflection coefficient (in dB), radiation gain (in dB), radiation efficiency (%), directivity (dB) and E-plane & H-plane far field radiation pattern.

## 3 Results and interpetation

In this design, the antenna is radiating at four different bands of the operating spectrum and thus showing a wide bandwidth of 410 MHz (3.62 GHz–4.03 GHz), 1210 MHz (4.17 GHz–5.38 GHz), 590 MHz (5.57 GHz–6.16 GHz) and 410 MHz (6.23 GHz–6.64 GHz) with a value of reflection coefficient to be -39 dB, -39.06 dB, -50.42 dB and -12.45 dB, thus making it a suitable candidate for the multiband operations in the mid-band 5G applications. The radiation gain (in dB) of an antenna depicts the power that can be transmitted in the direction of maximum radiation with reference to an isotropic antenna. In this proposed antenna design, the maximum gain extracted is 7.6 dB, 6.7 dB, 9.3 dB and 7.4 dB which makes it as a suitable candidate to be used for high gain applications. In this design, the radiation efficiency is extracted to be 82.27%, 83.27%, 79.87% and 70.04% respectively in the four bands of operation which is quite a good value. Further, the second characteristic of an antenna that is directivity is visualized which accounts for the directional property of an antenna. In case of omni-directional antennas, the value of directivity reduces to 1 (0 dB) as it radiates equally in all directions. In this design, the maximum directivity of about 8.4 dB, 7.5 dB, 9.9 dB and 8.6 dB in the four different frequency bands of operation is achieved which is very good for an antenna to be operated in the sub-6 GHz frequency spectrum. The Fraunhofer region on the E-plane and H-plane are made up of one fundamental projection and different side projections (also called as lobes) which is analyzed by measuring the value of half-power beamwidth. As the radiation is not so effective in the fourth band of operation i.e. ranging from 6.23 GHz to 6.64 GHz, hence the measurement analysis of the antenna is done only in the three effective radiating bands of operation. The value of half-power beamwidth for the three effective radiating bands of operation is found to be 78.9^0^, 61.2^0^ and 51.2^0^. The effect of the variation of the parameters on the reflection coefficient (dB) is examined and are given in Table 2, Table 3, Table 4 and Table 5. The parameter values are evaluated from the mathematical formulations as elaborated in the equations ranging from (1) to (13) and further optimizations are performed.

**Table 2:** Parametric analysis of the LTSA (L_TSA_ and W_TSA_).

Parameter	Proposed design values	Variation λ_m_	Resonant Frequency (GHz) in three bands of operation			Impedance Bandwidth (MHz)			Reflection Coefficient (dB) in the three frequency bands of operation		
Length of tapered slot antenna (L_TSA_)	1.76 λ_m_	1.60 λ_m_	3.82	5.17	5.98	470	1270	610	-41.45	-29.30	-31.63
		1.63 λ_m_	3.77	5.05	5.90	400	1180	580	-41.87	-52.12	-36.66
		1.65 λ_m_	3.77	5.03	5.87	410	1210	610	-42.24	-46.94	-38.83
		1.68 λ_m_	3.40	4.81	6.33	130	2300	380	-17.48	-32.39	-19.59
		1.69 λ_m_	3.41	4.80	6.33	140	2220	420	-18.56	-28.03	-21.25
		1.72 λ_m_	3.41	4.03	5.67	130	1480	560	-18.60	-30.69	-32.98
Width of tapered slot antenna (W_TSA_)	1.28 λ_m_	1.12 λ_m_	3.80	5.11	5.97	350	1160	510	-24.86	-42.39	-32.68
		1.15 λ_m_	3.77	5.03	5.90	370	1180	590	-32.65	-34.23	-23.41
		1.18 λ_m_	3.76	5.01	5.87	380	1140	520	-42.20	-38.49	-43.60
		1.24 λ_m_	3.76	5.01	5.86	400	1190	650	-33.13	-38.77	-31.75
		1.27 λ_m_	3.73	4.53	5.85	400	1160	670	-32.81	-47.40	-24.92
		**1.30**λ_m_	**3.76**	**4.57**	**5.86**	**420**	**1210**	**690**	**-27.97**	**-53.35**	**-24.51**

**Table 3:** Parametric analysis of the LTSA (D_S_, S_OW_ and S_OL_).

Parameter	Proposed design values	Variation λ_m_	Resonant Frequency (GHz) in three bands of operation			Impedance Bandwidth (MHz)			Reflection Coefficient (dB) in the three frequency bands of operation		
Circular slot diameter (D_S_)	0.39 λ_m_	0.32 λ_m_	3.85	4.97	6.38	870	410	850	-23.65	-13.40	-25.60
		0.34 λ_m_	3.82	-	5.98	1510	-	980	-27.54	-	-24.02
		0.35 λ_m_	3.80	-	5.92	1630	-	1110	-34.63	-	-34.36
		0.38 λ_m_	3.72	-	5.80	360	-	1780	-37.43	-	-28.85
		0.40 λ_m_	3.67	-	5.75	360	-	1660	-35.29	-	-24.72
		0.41 λ_m_	3.62	-	5.67	370	-	1530	-29.69	-	-22.17
Slot opening width (S_OW_)	0.06 λ_m_	0.01 λ_m_	-	-	5.61	-	-	1220	-	-	-19.58
		0.03 λ_m_	-	-	5.68	-	-	2280	-	-	-27.79
		**0.04**λ_**m**_	**3.78**	**5.06**	**5.77**	**320**	**1230**	**520**	**-21.10**	**-24.60**	**-38.91**
		**0.07**λ_**m**_	**3.73**	**4.97**	**5.96**	**270**	**870**	**500**	**-19.49**	**-26.66**	**-29.43**
		0.09 λ_m_	3.70	4.95	-	250	780	-	-14.32	-19.89	-
		0.10 λ_m_	3.67	4.93	-	170	610	-	-11.49	-14.99	-
Slot opening length (S_OL_)	0.32 λ_m_	0.27 λ_m_	3.76	5.02	5.86	410	1210	610	-38.13	-35.94	-42.72
		0.28 λ_m_	3.77	5.02	5.87	390	1170	580	-37.59	-38.93	-45.62
		0.31 λ_m_	3.77	5.02	5.87	390	1170	580	-37.59	-38.93	-45.62
		0.33 λ_m_	3.77	5.02	5.87	390	1170	580	-37.59	-38.93	-45.62
		0.34 λ_m_	3.77	5.02	5.87	390	1170	580	-37.59	-38.93	-45.62
		0.36 λ_m_	3.77	5.02	5.87	390	1170	580	-37.59	-38.93	-45.62

**Table 4:** Parametric analysis of the LTSA (T_L_, T_AW_ and T_OW_).

Parameter	Proposed design values	Variation λ_m_	Resonant Frequency (GHz) in three bands of operation			Impedance Bandwidth (MHz)			Reflection Coefficient (dB) in the three frequency bands of operation		
Tapered length (T_L_)	1.02λ_m_	0.90 λ_m_	2.43	-	5.53	290	-	480	-12.18	-	-12.13
		**0.93**λ_**m**_	**3.72**	**5.01**	**5.86**	**350**	**1070**	**560**	**-25.03**	**-28.71**	**-26.03**
		**1.00**λ_**m**_	**3.81**	**4.50**	**5.86**	**400**	**1210**	**460**	**-36.44**	**-54.28**	**-24.92**
		1.03 λ_m_	-	4.43	-	-	2370	-	-	-36.53	-
		1.06 λ_m_	-	4.37	-	-	2320	-	-	-49.07	-
		1.09 λ_m_	3.93	-	5.77	990	-	1170	-29.09	-	-27.87
Tapered antenna width (T_AW_)	0.06 λ_m_	0.03 λ_m_	3.77	5.02	5.87	390	1160	570	-42.35	-33.72	-56.71
		0.04 λ_m_	3.73	4.98	5.86	370	1100	540	-38.36	-32.54	-38.03
		0.07 λ_m_	3.77	5.02	5.87	410	1170	590	-34.48	-56.97	-38.11
		0.09 λ_m_	3.76	5.00	5.87	420	1190	640	-32.42	-40.04	-30.90
		0.10 λ_m_	3.76	5.03	5.87	420	1260	650	-29.79	-49.97	-32.46
		**0.12**λ_**m**_	**3.76**	**5.03**	**5.87**	**430**	**1270**	**660**	**-27.66**	**-33.90**	**-30.63**
Tapered opening width (T_OW_)	1.15 λ_m_	**1.03**λ_**m**_	**3.76**	**5.00**	**5.87**	**420**	**1190**	**640**	**-32.42**	**-40.04**	**-30.90**
		1.06 λ_m_	3.77	5.02	5.87	410	1170	590	-34.48	-56.97	-38.11
		1.12 λ_m_	3.73	4.98	5.86	370	1100	540	-38.36	-32.54	-38.03
		1.15 λ_m_	3.77	5.02	5.87	390	1160	570	-42.35	-33.72	-56.71

**Table 5:** Parametric analysis of the LTSA (H, G_W_ and G_L_).

Parameter	Proposed design values	Variation λ_m_	Resonant Frequency (GHz) in three bands of operation			Impedance Bandwidth (MHz)			Reflection Coefficient (dB) in the three frequency bands of operation		
Substrate height (H)	0.024 λ_m_	0.012 λ_m_	-	4.67	-	-	440	-	-	-30.42	-
		0.048 λ_m_	-	-	-	-	-	-	-	-	-
		0.072 λ_m_	-	-	-	-	-	-	-	-	-
		0.096 λ_m_	-	-	-	-	-	-	-	-	-
Ground width (G_W_)	0.08 λ_m_	0.03 λ_m_	4.07	-	5.37	170	-	1030	-11.08	-	-36.92
		**0.04**λ_**m**_	**4.00**	**5.02**	**6.63**	**340**	**1030**	**1040**	**-22.15**	**-24.86**	**-32.91**
		**0.06**λ_**m**_	**3.87**	**5.06**	**5.93**	**400**	**1100**	**520**	**-35.58**	**-29.19**	**-21.03**
		**0.09**λ_**m**_	**3.68**	**5.00**	**5.82**	**290**	**1000**	**430**	**-22.66**	**-54.85**	**-22.04**
		0.10 λ_m_	3.63	4.98	5.78	240	900	340	-17.23	-32.81	-17.09
		0.12 λ_m_	3.58	4.98	5.76	190	840	280	-14.20	-26.60	-15.04
Ground Length (G_L_)	0.67 λ_m_	0.59 λ_m_	4.06	-	6.13	1270	-	840	-12.82	-	-21.10
		0.60 λ_m_	3.97	-	5.98	1520	-	970	-17.77	-	-22.44
		0.62 λ_m_	3.87	-	5.91	1680	-	1110	-25.24	-	-27.31
		**0.65**λ_m_	**3.67**	**4.58**	**5.83**	**350**	**1030**	**530**	**-37.69**	**-34.69**	**-24.12**
		0.66 λ_m_	3.57	4.61	5.83	330	850	450	-39.79	-23.11	-19.22
		0.68 λ_m_	3.48	4.61	5.82	330	710	390	-35.63	-18.96	-16.28

**Figure 4:**
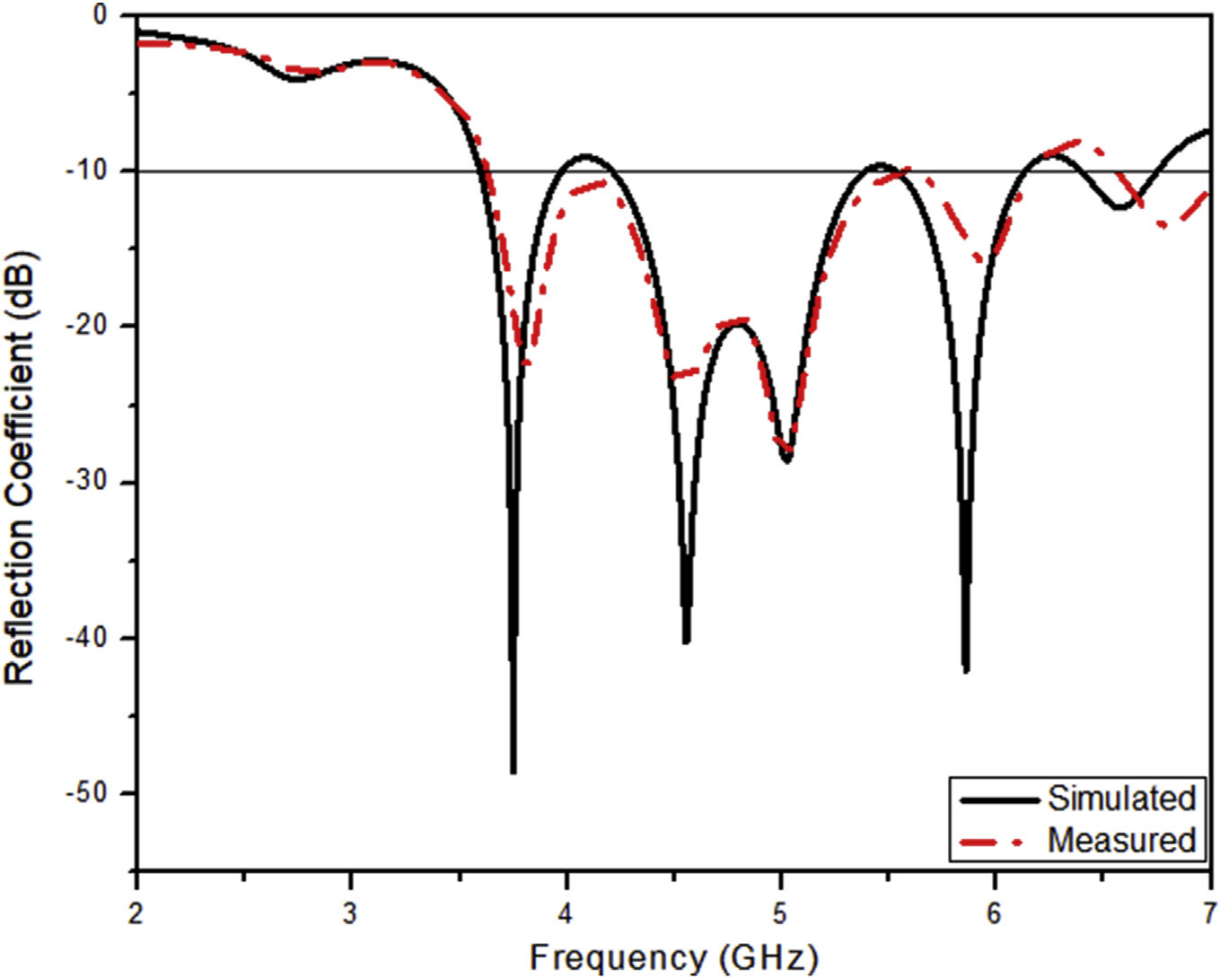
Reflection coefficient of the simulated and fabricated LTSA.

The comparative illustration of the reflection coefficient curve versus the frequency of operation for the designed prototype and the simulated antenna is shown in the Figure 4. As seen in the figure, it is interpreted that both the structures are resonating at four operating frequency bands within the desired operating range but the minimum reflection coefficient values are changed in form of -21 dB, -28 dB, -15 dB and -13 dB as compared to the values of the simulated antenna which are -39 dB, -39.06 dB, -50.42 dB and -12.45 dB respectively. This curve helps us to visualize the multiband operation in sub-6 GHz frequency spectrum giving broadband operation.

**Figure 5:**
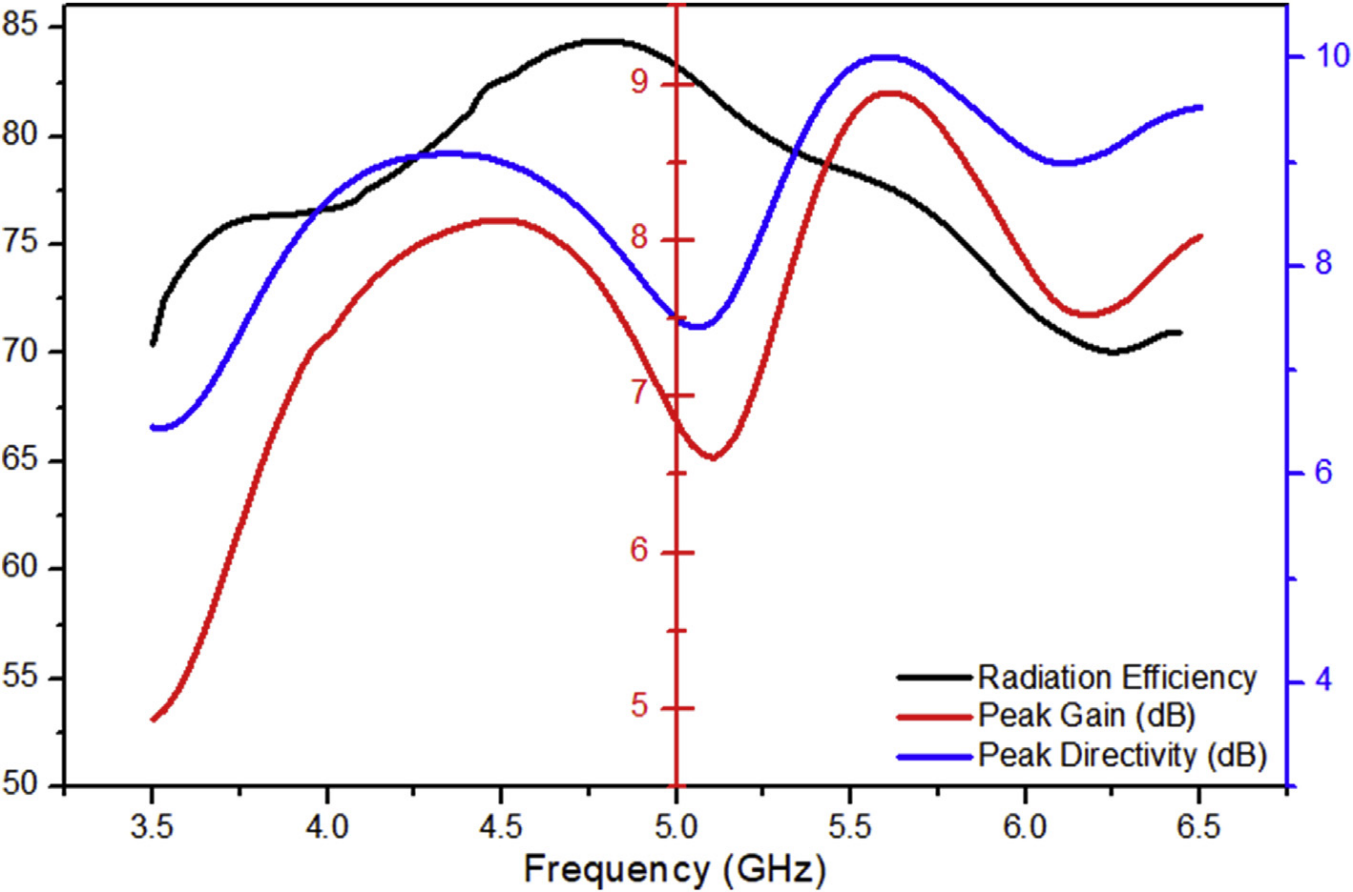
Gain, directivity and radiation efficiency of the LTSA.

The proposed antenna exhibits a high gain and directivity at all the resonating frequencies inside the operating band of mid-band 5G spectrum. The proposed design shows a good value of radiation efficiency which signifies that the amount of power radiated at each operating frequency. The graph shows the variation of the peak gain, peak directivity and the radiation efficiency with respect to frequency is shown in the Figure 5.

The proposed antenna radiates effectively only within the three bands of operation i.e. 3.62 GHz–4.03 GHz, 4.17 GHz–5.38 GHz and 5.57 GHz–6.16 GHz. Half power beamwidth i.e. -3 dB beamwidth at various frequencies in the different bands of operation are depicted in the Table 6.

**Table 6:** 3 dB beamwidth at of the LTSA.

Band of operation	Frequency of operation (in GHz)	-3dB Beamwidth (in degrees)	Frequency of operation (in GHz)	-3dB Beamwidth (in degrees)
(3.62 GHz-4.03 GHz)	3.62	86.37	3.72	83.06
	3.78	81.04	3.86	79.02
	3.92	78.01	4.03	77.46
(4.17 GHz-5.38 GHz)	4.17	77.32	4.91	67.02
	5.06	57.06	5.25	47.99
	5.35	48.61	5.38	48.97
(5.57 GHz-6.16 GHz)	5.57	52.34	5.62	53.08
	5.80	53.41	6.00	48.40
	6.14	44.62	6.16	44.43

**Figure 6:**
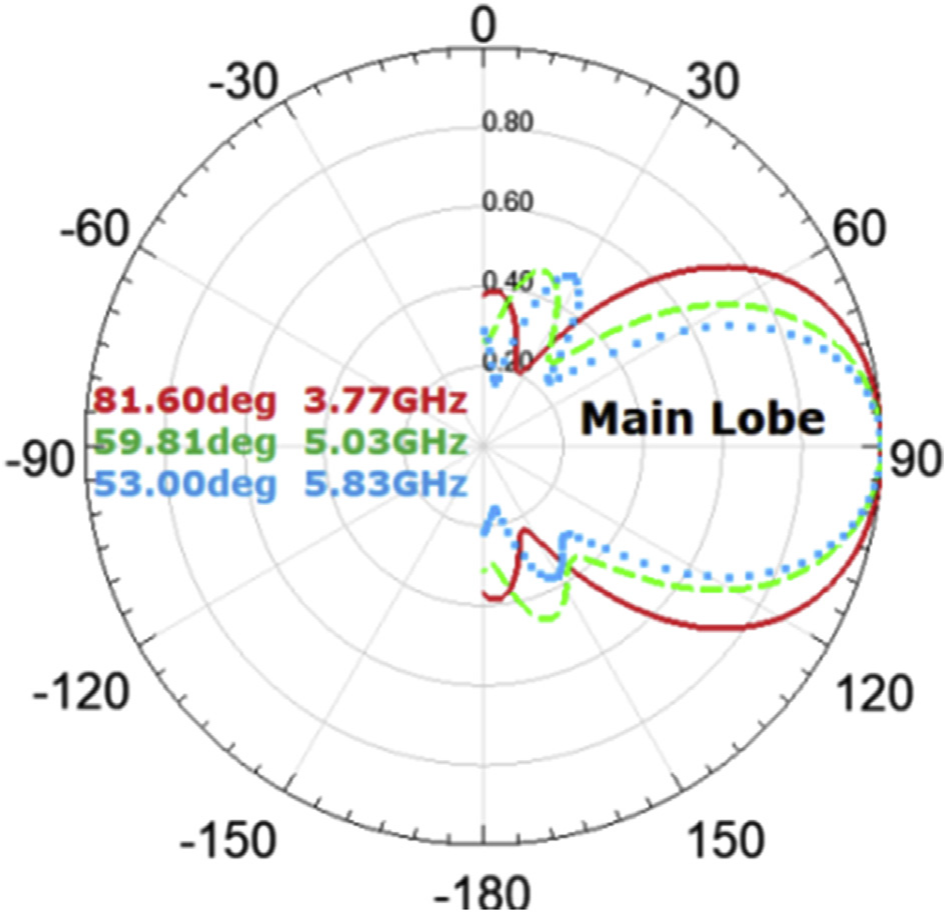
3-dB beamwidth of the LTSA at various frequencies.

The graphical illustration of the half-power beamwidth for mainly three effectively radiating bands of operation is further shown in Figure 6. The angle where the relative power exceeds 50% of the maximum output in the directional antennas is referred to as the half-power beamwidth. This is seen to be the most consistent and useful element of the antenna's transmission and it is closely tied to the directional antenna's gain.

**Figure 7:**
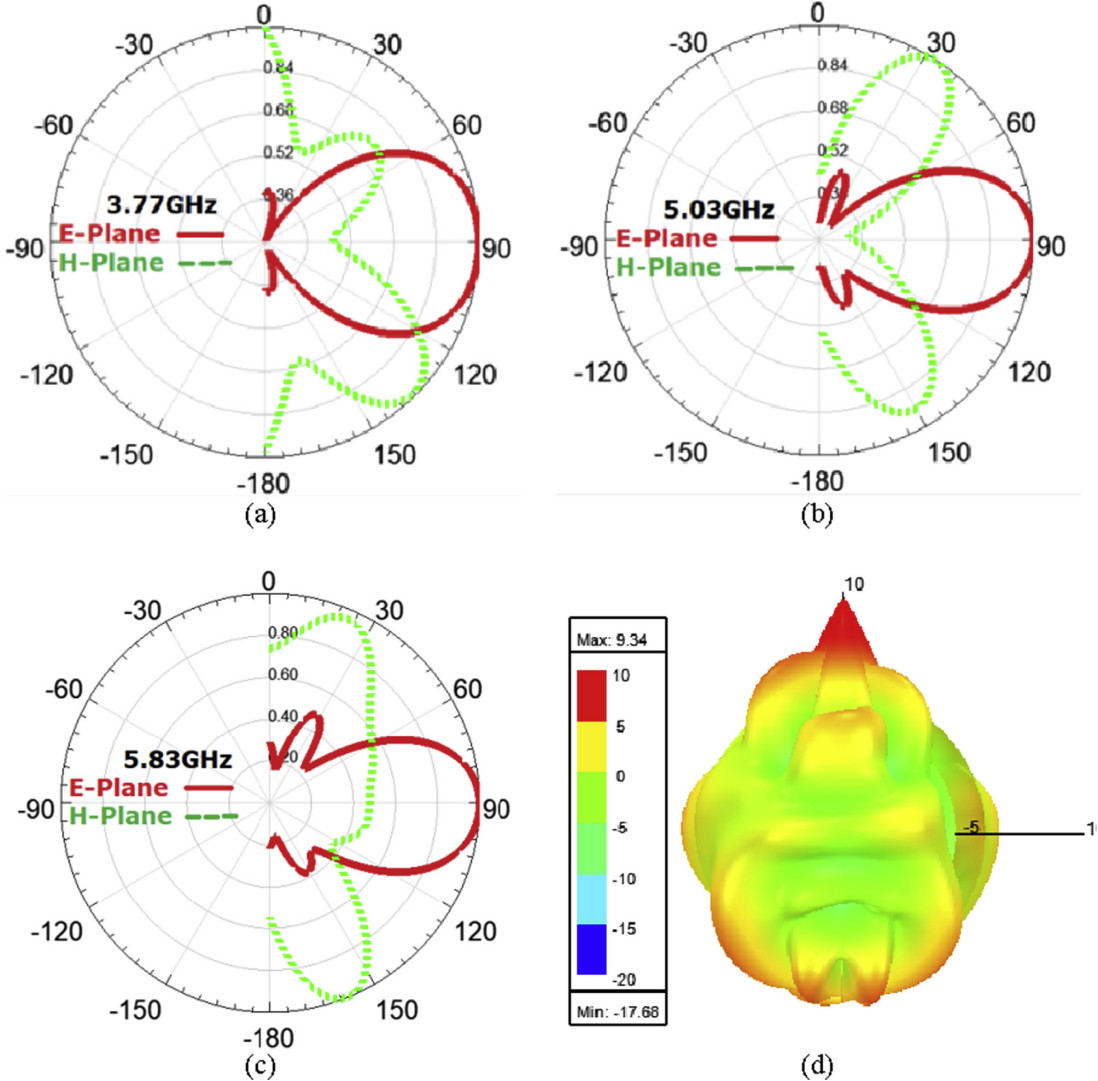
Radiation patterns, (a) at 3.77 GHz, (b) at 5.03 GHz, (c) at 5.83 GHz and (d) 3D plot.

The radiation pattern, often known as the antenna pattern, is a graphical depiction of the antenna's radiation qualities as a function of space. That is, the design of an antenna explains how the antenna radiates energy into space (or how it receives energy). It is critical to note that an antenna emits radiation in all directions; hence the antenna pattern is three-dimensional. However, it is usual to express this 3D pattern by using two planar patterns which are known as the primary plane patterns. These major plane patterns can be created by cutting two slices through the 3D pattern at the pattern's highest value or by precise observation. These primary plane designs are usually referred to as antenna patterns. The results are further analyzed by plotting the E-plane & H-plane radiation patterns (far field) in the Fraunhofer region are shown in Figure 7 (a), (b), (c) and (d).

The comparative analysis of the output characteristics from the proposed LTSA as resulted from the simulated and measured results is presented in the Table 7.

**Table 7:** Characteristics exhibited by the simulated LTSA and the LTSA prototype.

Characteristics	Values							
	Simulated				Measured			
Operating frequency band (GHz)	(3.62–4.03)	(4.17–5.38)	(5.57–6.16)	(6.23–6.64)	(3.64–4.08)	(4.15–5.40)	(5.58–6.16)	(6.51–7.32)
Resonant Frequency (GHz)	3.77	5.03	5.87	6.43	3.87	5.03	5.94	6.8
Impedance Bandwidth (MHz)	410	1210	590	410	440	1250	580	810
Reflection Coefficient (dB)	-49	-42.06	-44.42	-12.45	-21	-28	-15	-13
Radiation Efficiency (%)	84.27	83.27	79.87	70.04	79.82	81.42	77.46	68.67
Directivity (dBi)	8.4	7.5	9.9	8.6	8.3	7.2	9.6	8.48
Peak Radiation Gain (dB)	7.6	6.7	9.34	7.4	7.5	6.4	8.5	7.2

## 4 Evaluation of antenna performance for V2X systems

V2X communication set-up requires a priori knowledge of the path loss model to be utilized as it uses the wireless transmission links. The traditional channel model is created based on the distribution features of the metropolitan blocks scenario to be used in the V2V communication. The model is created for properly reciprocating the absorption and congestion of the signals which are used to study the viability of the 5G smart transportation resource allocation method [31]. In the vehicular communications, the shadowing concept maintains a normal distribution and is defined as in Eq. (14) given below:

(14) $$\text{n}-\text{s}\left(\text{t}\right)=\frac{1}{\sqrt{2\text{πσ}}}\exp\left(-\frac{{\left(\text{t}-\text{μ}\right)}^{2}}{2{\text{σ}}^{2}}\right)$$

where μ denotes the expectation value and σ represents the standard deviation. Also, $\sigma^{2}$ gives the information about the variance of the distribution.

The sub-6 GHz frequency bands are used for 5G NR-V2V connectivity. In the sub-6 GHz spectrum, the WINNER + B1 routing protocol may be applied in the metropolitan block scenario [32]. The antennae elevation of the mobile terminal is expected to be significantly smaller than the elevation of the neighboring buildings. The wireless transmission comprises of the light-of-sight (LOS) and the non-line-of-sight (NLOS) channels. The route loss model in the sub-6 GHz frequency can be described by using the WINNER routing protocol as shown in the Eq. (15) given below [32]:

(15) $$\text{PL ​}\left(\text{dB}\right)={\text{P ​log}}_{10}\left(\text{r}\right)+\text{Q}+{\text{R ​log}}_{10}\left({\text{f}}_{\text{c}}/5\right)$$

where r denotes the range that occurs between the transmitting and the receiving ends, and fc represents the frequency of operation. The constant P denotes the path loss index, Q represents the intercept coefficient and R signifies the path loss frequency dependence coefficient. The path loss model for a line of sight in the vehicular wireless communication is represented in the Eqs. (16), (17), (18), and (19) as given below [32]:

(16) $$\text{PL ​}\left(\text{dB}\right)=\left\{ \begin{array}{l} 22.7{\text{ ​log}}_{10}\left(3\right)+27.0+20.0{\text{ ​log}}_{10}\left({\text{f}}_{\text{c}}\right),\text{ ​ ​r}\leq 3\\ 22.7{\text{ ​log}}_{10}\left(\text{r}\right)+27.0+20.0{\text{ ​log}}_{10}\left({\text{f}}_{\text{c}}\right),3<r\leq {\text{r}}_{\text{x}}\\ 40.0{\text{ ​log}}_{10}\left(\text{r}\right)+7.56-17.3{\text{ ​log}}_{10}\left({{\text{h}}_{\text{Tx}}}^{\text{′}}\right)\text{,}\text{ ​ r}>{\text{r}}_{\text{x}}-17.3{\text{ ​log}}_{10}\left({{\text{h}}_{\text{Rx}}}^{\text{′}}\right)+2.7{\text{ ​log}}_{10}\left({\text{f}}_{\text{c}}\right),\text{ ​ ​ ​ ​ ​r}>{\text{r}}_{\text{x}} \end{array}\right.$$

where,

(17) $${\text{r}}_{\text{x}}=4{{\text{h}}_{\text{Tx}}}^{\text{′}}{{\text{h}}_{\text{Rx}}}^{\text{′}}{\text{f}}_{\text{c}}/\text{c}$$

(18) $${{\text{h}}_{\text{Tx}}}^{\text{′}}={\text{h}}_{\text{Tx}}-1$$

(19) $${{\text{h}}_{\text{Rx}}}^{\text{′}}={\text{h}}_{\text{Rx}}-1$$

The term c denotes the velocity of light having the value of $3\times 10^{8}$ m/sec, ${\text{h}}_{\text{Tx}}$ and ${\text{h}}_{\text{Rx}}$ denotes the effective height of the transmitter and the receiver respectively. Also, the path loss (PL) model of the NLOS is described in the Eq. (20) as given below [32]:

(20) $$\text{PL}=\min\left\{\text{PL ​}\left({\text{r}}_{1},{\text{r}}_{2}\right),\text{PL ​}\left({\text{r}}_{2},{\text{r}}_{1}\right)\right\}$$

where r_1_ denotes the vertical range from the transmitting end to the centre of the street, and r_2_ represents the range from the centre of the street to the receiving end. The term PL (r_k_,r_l_) k∈1,2,l∈1,2 can be given as illustrated in the Eqs. (21) and (22) as:

(21) $$\text{PL ​}\left({\text{r}}_{\text{k}},{\text{r}}_{\text{l}}\right)={\text{PL}}_{\text{LOS}}\left({\text{r}}_{\text{k}}\right)+17.3-12.5{\text{η}}_{0}+10{\text{η}}_{0}\;\log\left({\text{r}}_{\text{j}}\right)+3\;\log\left({\text{f}}_{\text{c}}\right)$$

(22) $${\text{η}}_{0}=\text{max}\left(2.8-0.002{\text{r}}_{\text{k}},1.84\right)$$

**Figure 8:**
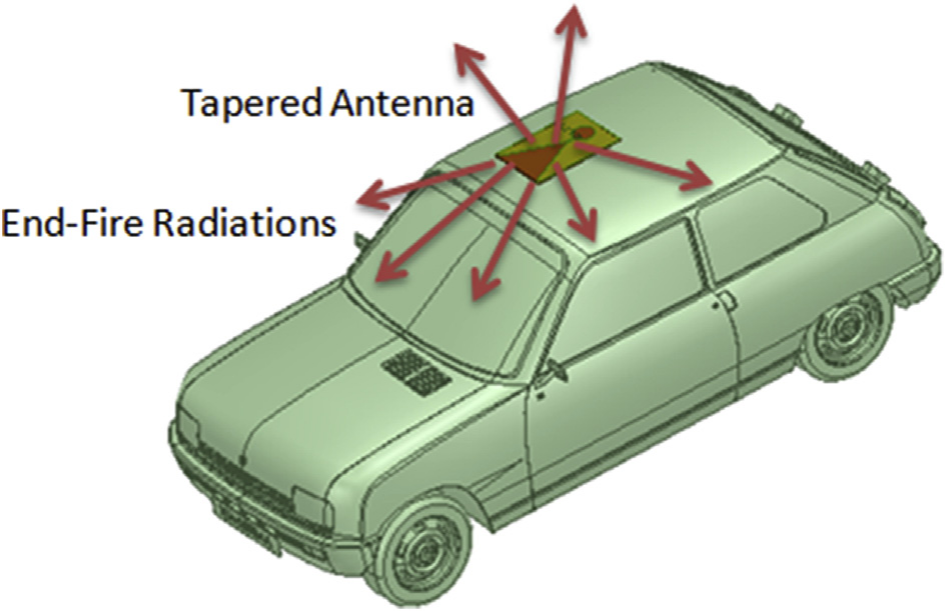
Placement of the LTSA on an automobile.

Another, important parameter to be taken into account is the received signal strength indicator (RSSI) which is an assessment of the energy contained in an intercepted radio wave in V2X communications. An end user of the V2X communication is typically unaware of the value of the RSSI which is attained. However, as the signal strength varies frequently and has an adverse effect on the wireless networking capability, hence the IEEE 802.11 devices frequently make the measurement available to the users. Its value is extracted as illustrated in the Eq. (23) as given below [32]:

(23) $$\text{RSSI}=10\;\log\left({\text{P}}_{\text{TRc}}\right)-\text{ ​PL ​}\left(\text{dB}\right)+\text{ ​Ga}\left(\text{θ},\text{ф}\right)$$

where the term P_TRc_ is the transmitted power of the transreceivers, PL (dB) denotes the path loss over the range of communication, and the term Ga (θ,φ) represents the gain of an antenna. The installation of the vehicular antenna plays a vital role in the enhanced functionality of the automotive antenna systems [33, 34]. Path loss models plot the transmission loss in between transmission and receiving antennas as a function of transmission losses as well as other variables which affect the performance. To predict the performance degradation, some models also incorporate various aspects of the topographical profile, while others only consider the carrier signal and distance between the transmitter and the receiver antennas. In case, of vehicular communication, the antenna is mounted on the roof top and the signal is received from striking through the different paths of propagation. Understanding and characterizing the propagation characteristics of the vehicular networks is crucial for satisfying the requirements of safety and non-safety applications in the vehicular networks, especially in the environments with high road traffic density and endpoint mobility. The research on the design of vehicular antennas can be extended for predicting the route loss model which can be realized based on the narrowband channel measurements to be conducted at sub-6 GHz 5G NR spectrums. Since the electromagnetic field distribution can be severely impacted by the shape and the material of the vehicle body and hence the antenna's placement is severely restricted due to aesthetic considerations as imposed by the vehicle industry and due to the proximity of other electrical equipment [35]. The designed antenna is placed at the centre in the front end of the roof above the front main glass of the vehicle due to its maximum coverage and minimum interference with the diffractions as depicted in Figure 8. The tapered antenna is placed on the roof at the front end of the automobile. The roof of an automobile is chosen by most of car manufacturers because the roof of an automobile is high above the ground and is always unobstructed. It helps in achieving a good reception rate in almost all the directions of an antenna. Antennas for automobiles must meet certain requirements to get a decent reception. First and foremost, while this may seem self-evident, the antenna must be able to transmit or receive signals from all the directions around the vehicle which requires a high amount of gain and directivity. If a single antenna isn't enough to meet this criterion, then an antenna array (maybe thought off). In general, an antenna on a vehicle should be as high above the ground as practicable as it can receive and broadcast better the more it is situated above ground. The antenna must then as simple to be engraved into the vehicle. Further, the distance between the transmitter and the receiver within an automobile should be kept to a minimum, so that the incoming signals are not attenuated before being utilized. The antenna's surroundings might have a significant impact on its performance. As a result, the materials and the distances with other conductors present in the surroundings must be taken into account. As a matter of thumb, the antenna should be surrounded by a box that must be as good as three times the size of an antenna. Which implies that only the telephone or GPS antennas operating above 1 GHz finds their suitability.

**Figure 9:**
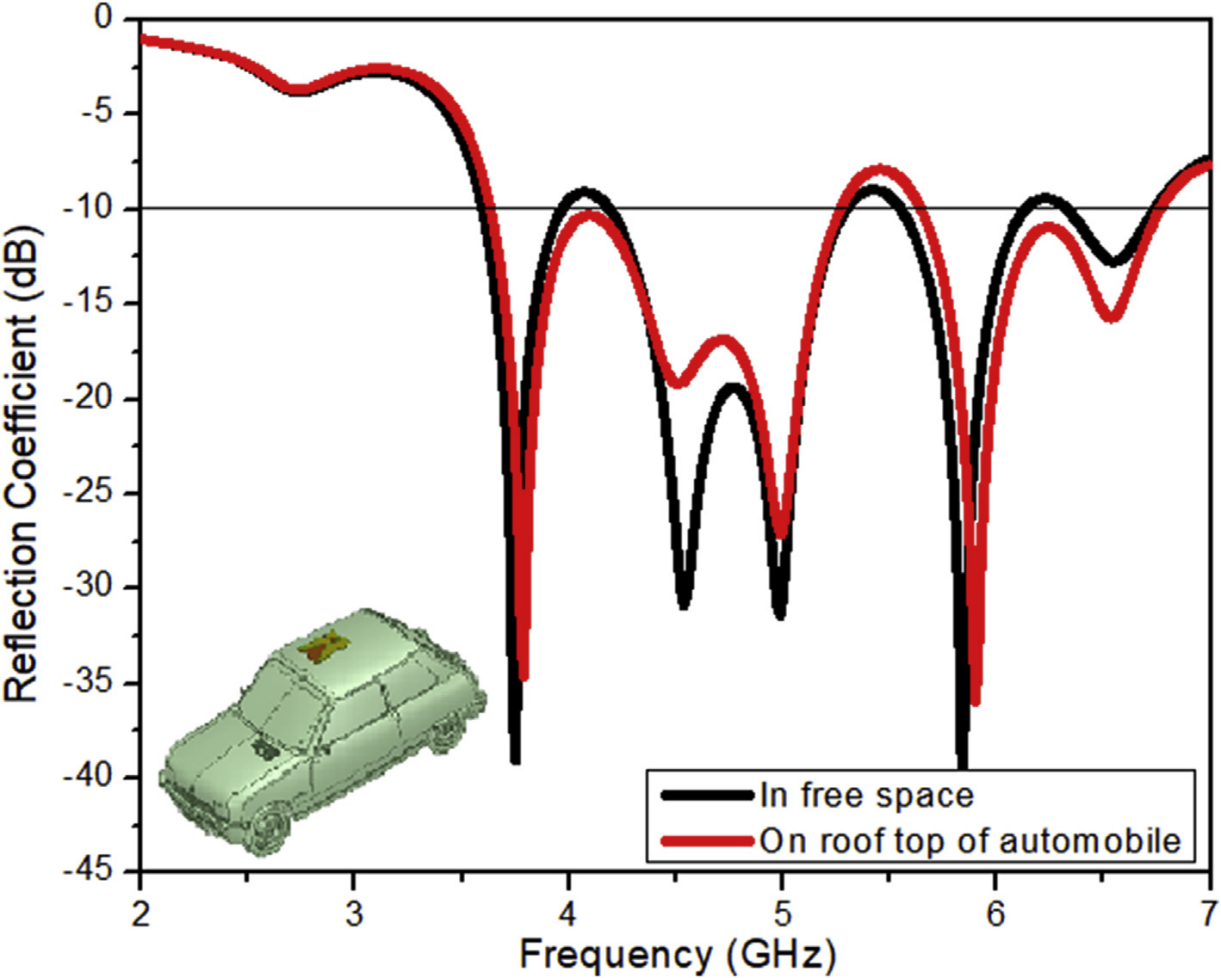
Reflection coefficient of the LTSA placed on the rooftop of an automobile.

**Figure 10:**
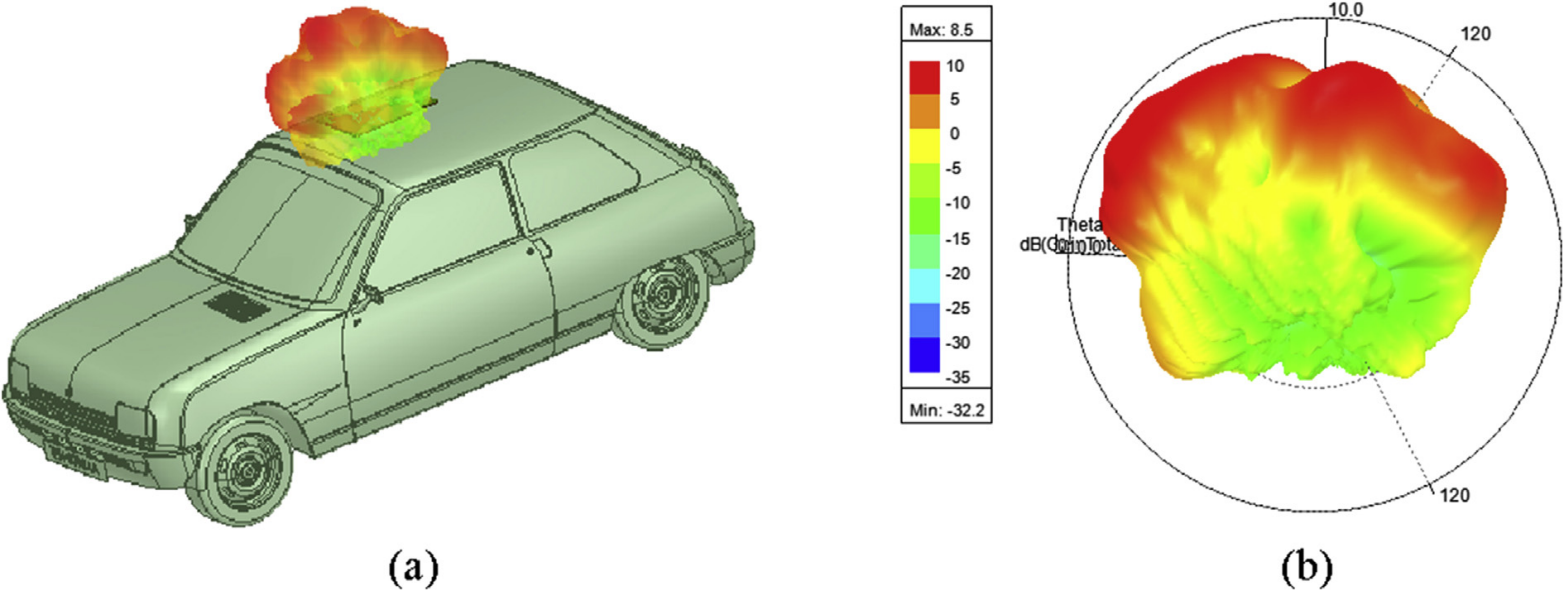
3d radiation pattern of the LTSA placed on the front end of the roof top of an automobile (a) pattern with automobile, (b) pattern with labels.

The tapered slot antenna as designed in the section 2 is hence placed on the top of the roof of an automobile and simulations are being performed. The variation in the radiation performance is retrieved from the comparative analysis of the reflection coefficient variation with the frequency of operation as shown in Figure 9. The variations in the reflection coefficient curve occur due to an increase in the shooting and bouncing rays (SBR) and because of the creeping wave excitation which occurs when an antenna is placed on the top of the metallic body of an automobile. There are some losses due to SBR but still, antenna manages to radiate effectively within the desired operating region as analyzed in the free space. When the designed tapered slot antenna is placed in the center of front roof as shown in the Figure 8, the retrieved electric field distributions are perfect, and can be an effective means in vehicle communication. The effective vertical radiation patterns depicting the amount of gain delivered by the designed antenna are shown in Figure 10.

It is clear from the Figure 10 that the amount of peak gain is reduced to 8.5 dB from the original value of 9.3 dB, when the antenna is placed on the vehicle. It may be due to the creeping wave losses and from the scattering that occurs from the shooting and bouncing rays striking the body of an automobile.

**Figure 11:**
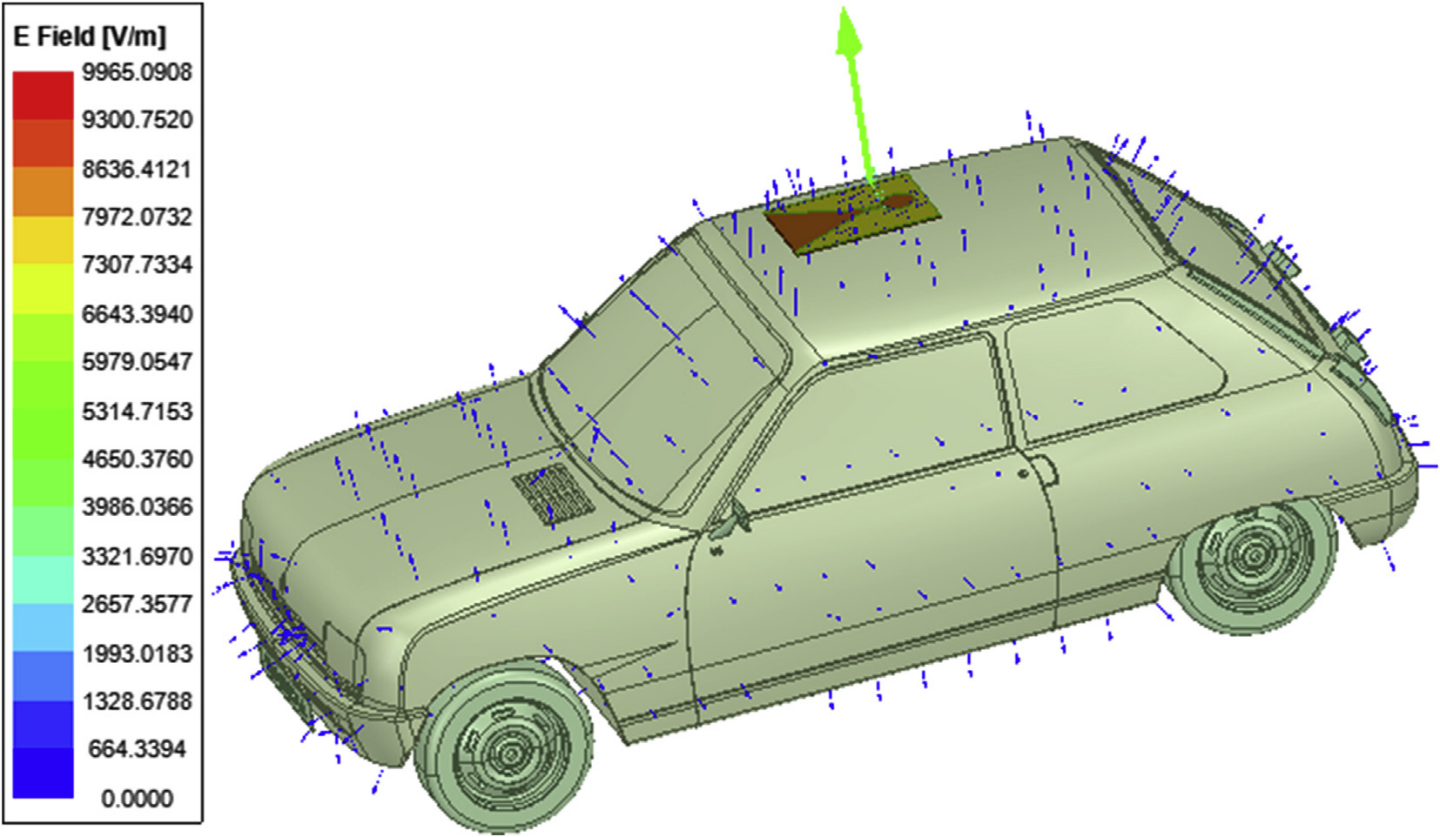
Electric field emitted from the LTSA placed on the roof top of an automobile and from the SBR of the vehicle.

**Figure 12:**
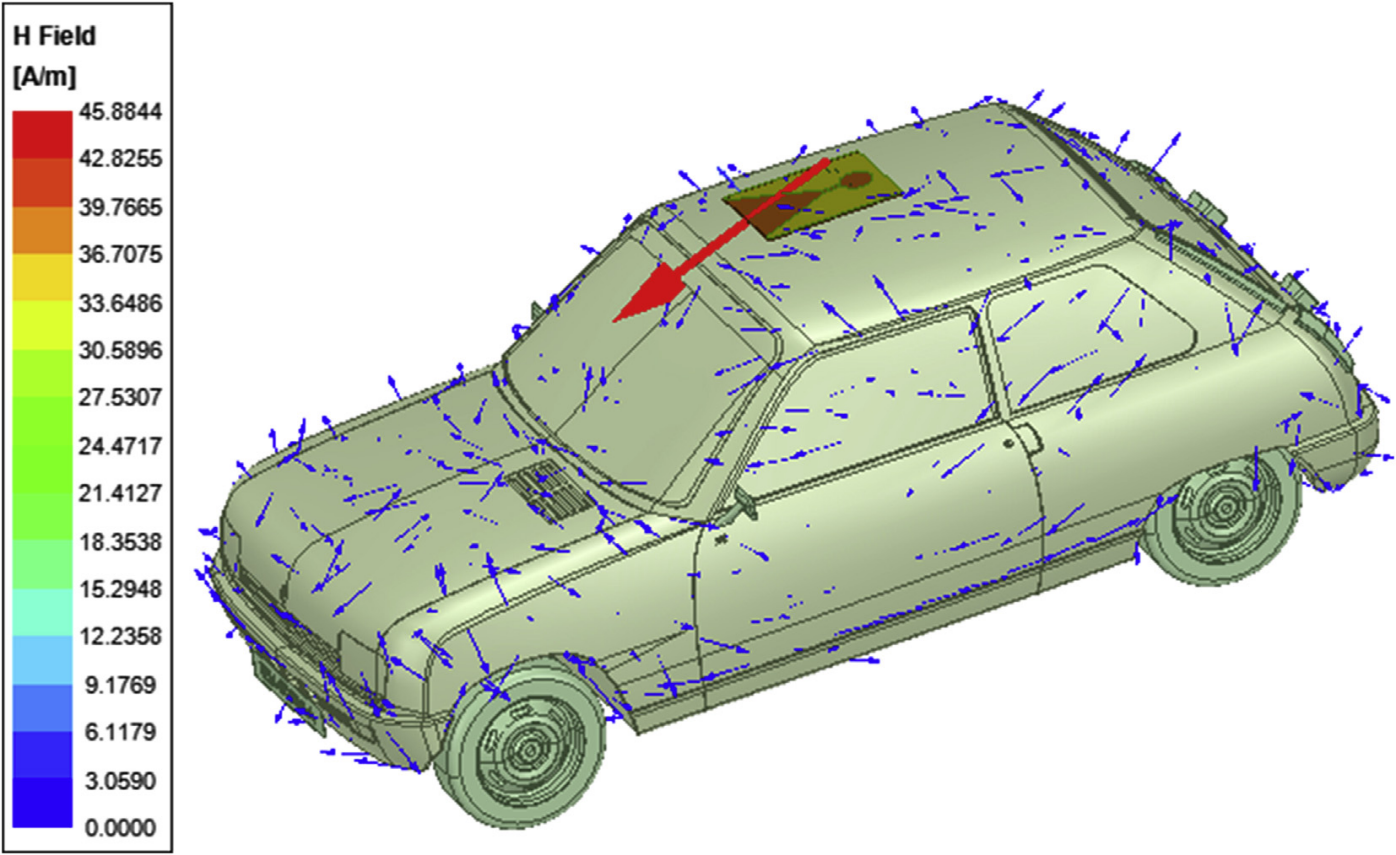
Magnetic field emitted from the LTSA placed on the roof top of an automobile and from the scattering phenomenon of the vehicle.

**Figure 13:**
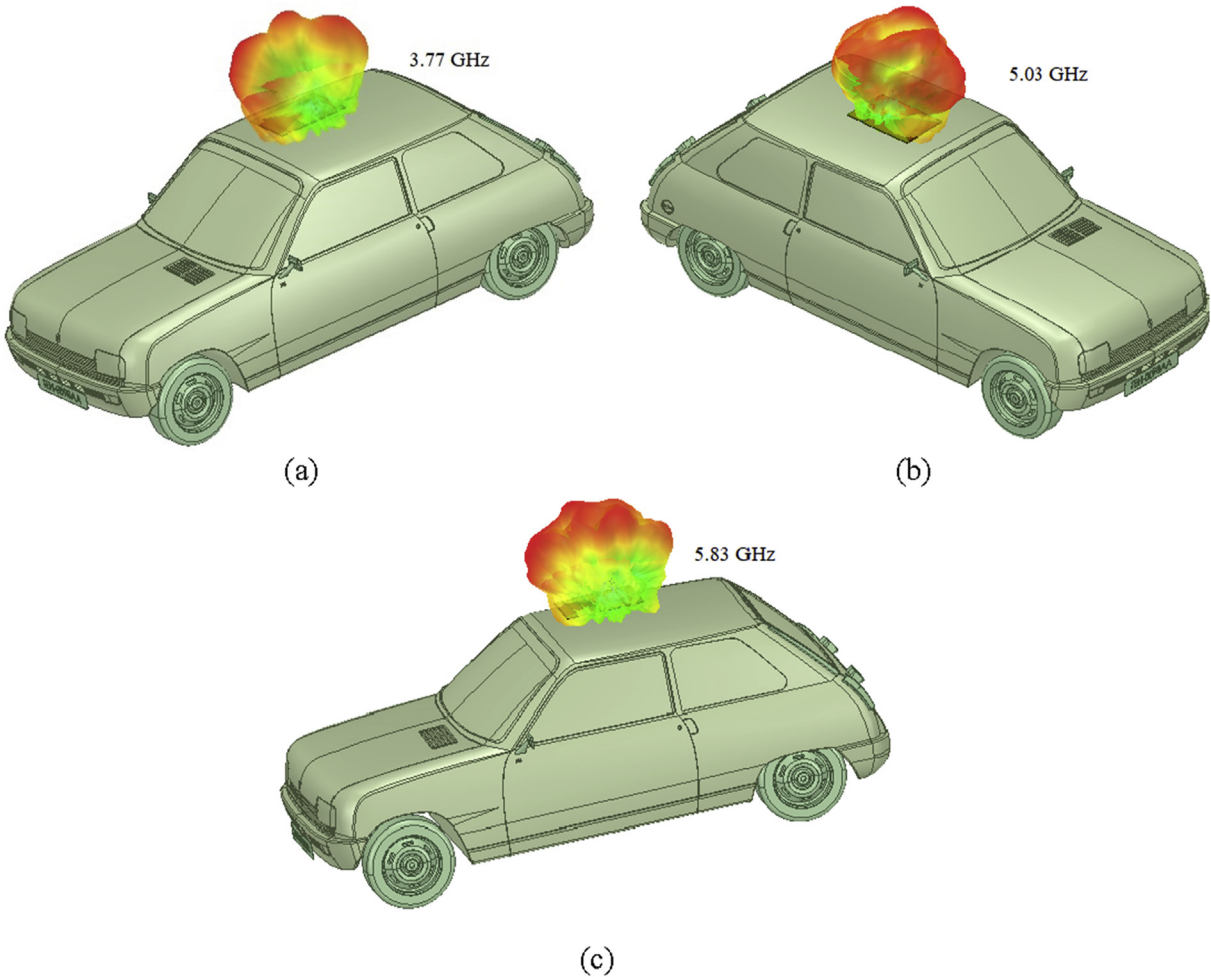
3-D pattern of the LTSA when placed on the roof top of an automobile at (a) 3.77 GHz, (b) 5.03 GHz, and (c) 5.83 GHz.

The vector electric field is plotted in Figure 11 indicating the directions of the electric field vector emitted from the body of an automobile and from the radiating patch aperture. The directions of the electric and the magnetic field vectors help in approximating the direction of an electromagnetic wave. The directions of the magnetic field vector for the patch antenna radiator and for the shooting and bouncing rays scattered from the body of an automobile is shown in Figure 12. The radiations being emitted from the designed tapered slot antenna radiator when placed on the rooftop of an automobile is depicted in the form of 3D polar plot as illustrated in Figure 13. It indicates that the designed antenna radiates effectively with directional behavior and thus providing it validity to be used in vehicular communication.

**Figure 14:**
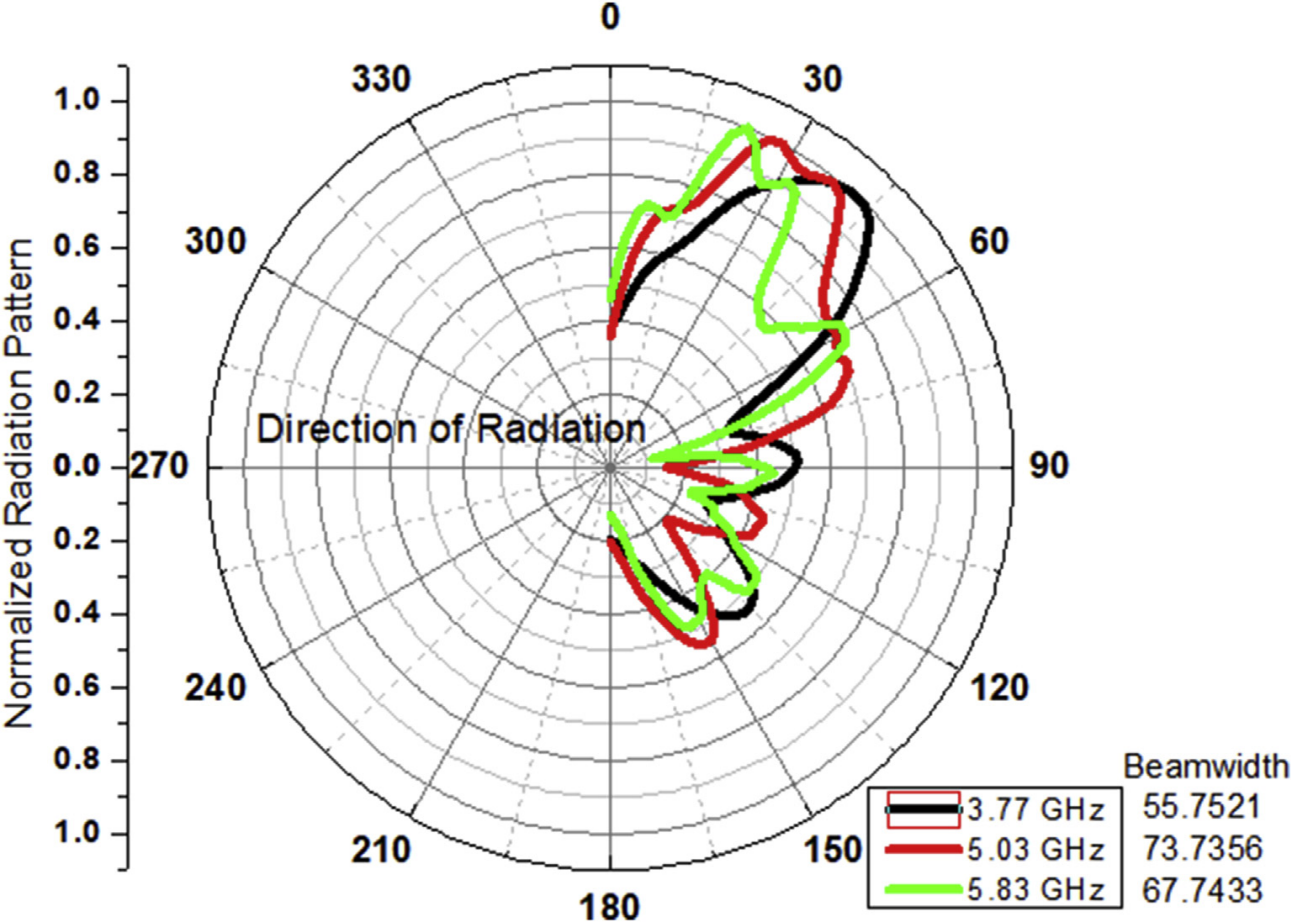
3-dB beamwidth of the LTSA when placed on the roof top of an automobile.

Against all the limitations of the scattering and the diffractions in the radiations being emitted by an automobile antenna, the beamwidth is sufficiently maintained to more than 50^o^ as indicated in Figure 14. A sufficient amount of the beamwidth is maintained after all the scattering and the diffractions which helps in increasing the quality of service (QoS) and helps in reduction in the backdrop.

The variations in the characteristics of the proposed LTSA design on placement over an automobile are depicted in the Table 8 as illustrated. It clearly indicates that due to scattering and diffractions of the signal rays from the conducting body of an automobile, there is slight shift in the radiating spectrum with reduction in the effective radiating parameters.

**Table 8:** Characteristics of the proposed LTSA when placed upon an automobile.

Characteristics	Values on vehicular placement		
Operating frequency band (GHz)	(3.63–4.07)	(4.17–5.38)	(5.58–6.83)
Resonant Frequency (GHz)	3.87	5.03	5.94
Impedance Bandwidth (MHz)	440	1210	1250
Reflection Coefficient (dB)	-36	-27	-36
Radiation Efficiency (%)	83.64	82.76	78.72
Directivity (dBi)	8.28	7.34	9.65
Peak Radiation Gain (dB)	7.4	6.3	8.53

**Figure 15:**
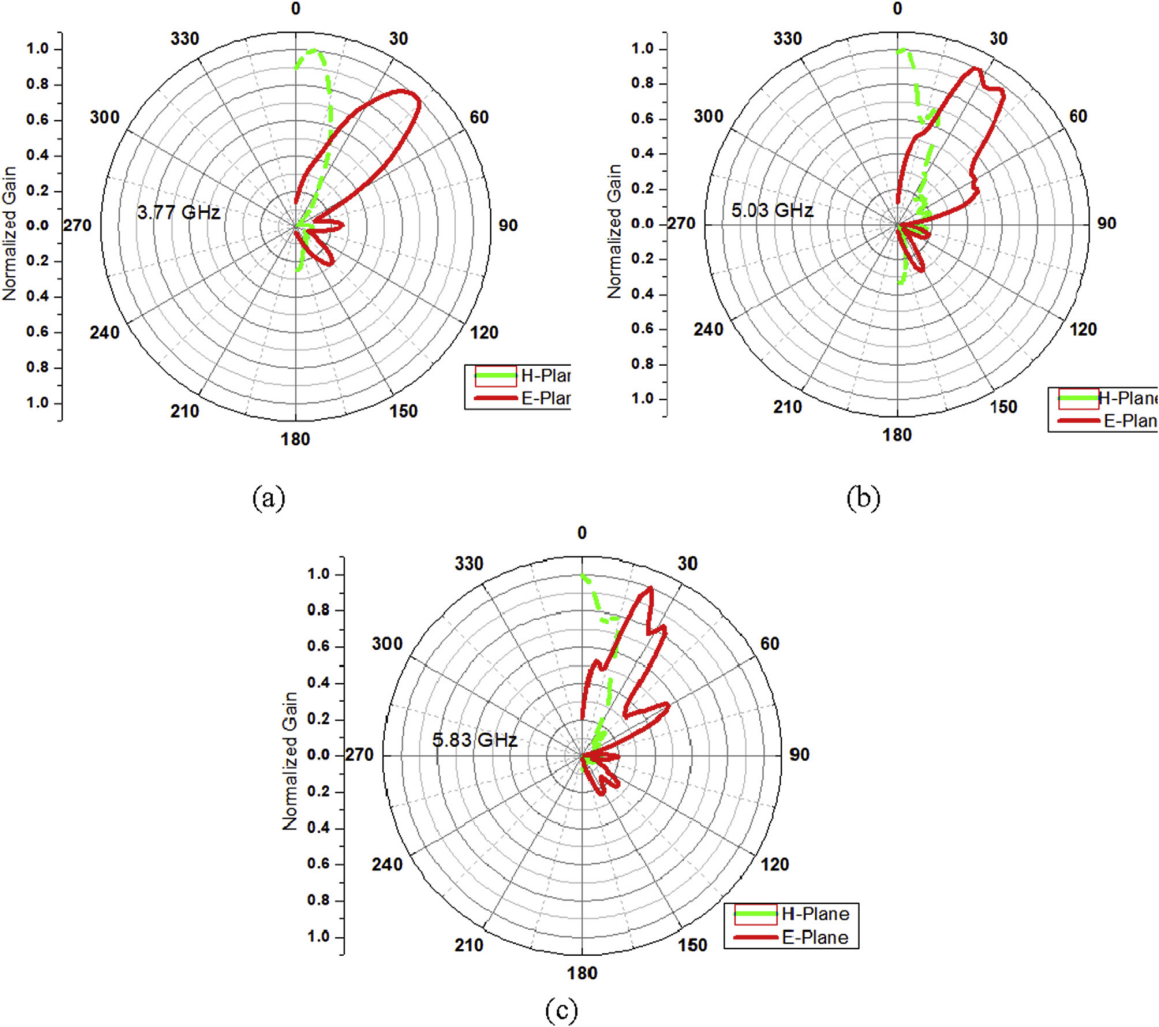
Normalized E-plane & H-plane radiation patterns of the LTSA on vehicular placement at (a) 3.77 GHz, (b) 5.03 GHz, and (c) 5.83 GHz.

The radiation phenomena is further analyzed by plotting the E-plane & H-plane radiation patterns in the Fraunhofer region on antenna placement at the roof top of an automobile to signify the electric and magnetic field orientations as illustrated in Figure 15(a), (b) and (c), respectively.

The comparative analysis of the proposed linear tapered slot antenna with the earlier designs proposed for dedicated application on the vehicular communication has been presented in Table 9 which justifies that this design is best among the reported state of art literature. The higher amount of gain retrieved is accounted for an additional rectangular slot which was incorporated in between the circular and the tapered slot structure. Also, the bandwidth is not affected and still, a multiband is achieved due to the microstrip to slot line progression feeding technique that is being used.

**Table 9:** Comparative analysis of the proposed LTSA placed on the rooftop of an automobile with the state-of-art designs.

Ref.	Parameters						
	Freq. Range (GHz)	Centre Freq. (GHz)	Patch Size	Operating Band	Substrate parameters	Bandwidth	Peak Gain (dBi)
[4]	1.19–1.21, 2.41–2.46, 5.56–5.70	3.4	$0.56{\text{λ}}_{\text{m}}\times 0.56{\text{λ}}_{\text{m}}$	Triple	$\varepsilon_{\text{r}}=4.4$, $\text{ ​H}=0.01{\text{λ}}_{\text{m}}$	10 MHz (1st), 15 MHz (2nd), 20 MHz (3rd)	∼6.3
[5]	5.29–5.32, 5.598–5.604, 5.797–5.802	5.5	$0.81{\text{λ}}_{\text{m}}\times 1.29{\text{λ}}_{\text{m}}$	Triple	$\varepsilon_{\text{r}}=3$, $\text{ ​H}=0.001{\text{λ}}_{\text{m}}$	8 MHz (1st), 8 MHz (2nd), 5MHz (3rd)	∼7.8
[36]	5.45–7.47	6.46	$0.30{\text{λ}}_{\text{m}}\times 0.38{\text{λ}}_{\text{m}}$	Single band	$\varepsilon_{\text{r}}=2.2,\text{ ​H}=0.03{\text{λ}}_{\text{m}}$	2020 MHz	∼7.8
[37]	24.3–41.95, 49.91–52.15	33.1, 51.03	10 × 13 mm^2^	Dual band	$\varepsilon_{\text{r}}=3.16,\text{ ​H}=0.254\text{mm}$	17.65 GHz, 2.24 GHz	∼6.59
[38]	21–23.5	22.25	$0.37{\text{λ}}_{\text{m}}\times 0.74{\text{λ}}_{\text{m}}$	Single band	$\varepsilon_{\text{r}}=4.3,\text{ ​H}=0.05{\text{λ}}_{\text{m}}$	2500 MHz	∼5.9
[39]	5.71–5.94	5.82	$2.03{\text{λ}}_{\text{m}}\times 1.55{\text{λ}}_{\text{m}}$	Single band	$\varepsilon_{\text{r}}=4.4,\text{ ​H}=0.03{\text{λ}}_{\text{m}}$	230 MHz	∼6.6
[40]	5.6–6.1	5.85	$0.78{\text{λ}}_{\text{m}}\times 0.78{\text{λ}}_{\text{m}}$	Single band	$\varepsilon_{\text{r}}=4.4,\text{ ​H}=0.03{\text{λ}}_{\text{m}}$	500 MHz	∼6.6
**Proposed vehicular** **LTSA**	3.62 GHz–4.03 GHz, 4.17 GHz–5.38 GHz, 5.57 GHz–6.16 GHz, 6.23 GHz–6.64 GHz	**3.77, 5.03,** **5.87,** **6.43**	$1.76\lambda_{m}\times 1.28\lambda_{m}$	**Quad band**	$\varepsilon_{r}=4.4$**,**$H=0.02\lambda_{m}$	**410 MHz (1**^**st**^**),** **1210 MHz (2**^**nd**^**),** **590 MHz (3**^**rd**^**)** **410 MHz (4**^**th**^**)**	∼9.3

## 5 Conclusion

A revolutionary automotive antenna operating over a broadband frequency range and based on the augmented Vivaldi antenna design is presented. The design of the antenna is the modified form of Vivaldi antenna which is in the form of a linear tapered slot structure. The fluctuation of the reflection coefficient with respect to the frequency of operation with and without the presence of a vehicle is used to characterize the radiations emitted by the antenna. The antenna is designed to have a high gain and a minimal loss. The presented LTPSA provides the maximum gain of 9.3 dB. The antenna's high directional radiation performance allows it to link mobile cellular networks and IoV systems successfully. As a result, the performance of the network coverage is increased by the presented antenna. The presented antenna might be widely utilized for the V2X mid-band 5G communication systems.

## Declarations

### Author contribution statement

Ankush Kapoor: Conceived and designed the experiments; Performed the experiments; Analyzed and interpreted the data; Contributed reagents, materials, analysis tools or data; Wrote the paper.

Pradeep Kumar, Ranjan Mishra: Conceived and designed the experiments; Analyzed and interpreted the data; Contributed reagents, materials, analysis tools or data; Wrote the paper.

### Funding statement

This research did not receive any specific grant from funding agencies in the public, commercial, or not-for-profit sectors.

### Data availability statement

Data included in article/supplementary material/referenced in article.

### Declaration of interests statement

The authors declare no conflict of interest.

### Additional information

No additional information is available for this paper.
